# Nanoclay Intercalation During Foaming of Polymeric Nanocomposites Studied in-Situ by Synchrotron X-Ray Diffraction

**DOI:** 10.3390/ma11122459

**Published:** 2018-12-04

**Authors:** Victoria Bernardo, Mikel Mugica, Saul Perez-Tamarit, Belen Notario, Catalina Jimenez, Miguel Angel Rodriguez-Perez

**Affiliations:** 1 Cellular Materials Laboratory (CellMat), Condensed Matter Physics Department, University of Valladolid, Paseo de Belen 7, 47011 Valladolid, Spain; mikel.mugica@fmc.uva.es (M.M.); saul.perez@fmc.uva.es (S.P.-T.); belen.notario@fmc.uva.es (B.N.); marrod@fmc.uva.es (M.A.R.-P.); 2 Helmholtz-Zentrum Berlin, Hahn Meitner Platz 1, 14109 Berlin, Germany; catalina.jimenez@helmholtz-berlin.de

**Keywords:** cellular nanocomposites, nanoclays, X-ray diffraction, synchrotron radiation, foams

## Abstract

The intercalation degree of nanoclays in polymeric foamed nanocomposites containing clays is a key parameter determining the final properties of the material, but how intercalation occurs is not fully understood. In this work, energy dispersive X-ray diffraction (ED-XRD) of synchrotron radiation was used as an in-situ technique to deepen into the intercalation process of polymer/nanoclay nanocomposites during foaming. Foamable nanocomposites were prepared by the melt blending route using low-density polyethylene (LDPE), polypropylene (PP), and polystyrene (PS) with surface treated nanoclays and azodicarbonamide (ADC) as the blowing agent. Foaming was induced by heating at atmospheric pressure. The time and temperature evolution of the interlamellar distance of the clay platelets in the expanding nanocomposites was followed. Upon foaming, interlamellar distances of the nanocomposites based on LDPE and PP increase by 18% and 16% compared to the bulk foamable nanocomposite. Therefore, the foaming process enhances the nanoclay intercalation degree in these systems. This effect is not strongly affected by the type of nanoclay used in LDPE, but by the type of polymer used. Besides, the addition of nanoclays to PP and PS has a catalytic effect on the decomposition of ADC, i.e., the decomposition temperature is reduced, and the amount of gas released increases. This effect was previously proved for LDPE.

## 1. Introduction

Polymeric foams are light-weight materials with an excellent strength-to-weight ratio and good thermal and sound insulation properties, having many applications in the insulating, packaging, construction, and automotive industries [1,2]. In particular, foamed polymer/clay nanocomposites are attractive for applications requiring high strength and lightweight and enhanced flammability resistance, among other properties [3,4,5]. Nanoclays have outstanding reinforcement efficiency in the production of cellular polymers due to their high surface-to-volume ratio [3,6]. The arrangement of the clays in the polymer has a strong influence on the properties [7,8] so that the particles can be aggregated (forming micron-sized clusters) or well dispersed (intercalated or exfoliated) [9,10]. There is extensive research on the effect of nanoclays’ addition in the properties of foams [11,12,13,14,15,16,17,18,19]. These works relate the properties with the state of dispersion/aggregation of the nanoclays in the nanocomposite bulk precursors before foaming. However, few works focus on the intercalation/exfoliation induced by the foaming process itself. 

Velasco et al. [20,21] found that polyethylene/hectorite foams showed a higher degree of intercalation than the initial material before foaming. The interlamellar spacing between clay platelets was measured using X-ray diffraction of the materials before and after foaming. The same methodology was employed by Laguna and coworkers [22] in polypropylene/montmorillonite foams. They found that after foaming, the interlamellar spacing had increased 1.4 times, which means a 40% increase in the intercalation degree compared to the solid material. These ex-situ results, however, did not provide insights into the mechanisms involved in this phenomenon. 

In our previous work [23], the effect of the foaming process on the intercalation of montmorillonite (MMT) nanoclays in low-density polyethylene (LDPE)/nanoclay nanocomposites containing different types of blowing agent was analyzed using in situ energy dispersive X-ray diffraction (ED-XRD) during the foaming experiments. We observed that, indeed, the foaming process enhanced the intercalation of the clays in LDPE with all the types of blowing agents used, but this effect was larger when the blowing agent was azodicarbonamide (ADC). Besides, the degree of intercalation was correlated with the expansion ratio: Larger expansions led to greater intercalations. Moreover, a reduction on the decomposition temperature of the blowing agent under the addition of clays was reported [23]. However, this work was focused on LDPE filled with a single type of organo-modified nanoclay (Cloisite C15A). Thereby, the influence of the clay surface modification and the nature of the polymeric matrix on the intercalation process and the decomposition kinetics of the blowing agent remained unclear. 

Therefore, in this work, three types of clays with different surface modifications and three thermoplastic polymeric matrices, namely LDPE, polypropylene (PP), and polystyrene (PS), were melt blended to make foamable nanocomposite precursor materials, using ADC as the blowing agent. The intercalation of the clays during foaming was followed by in situ ED-XRD experiments. This work aims to gain knowledge on the underlying mechanisms of the nanoclay intercalation during foaming.

## 2. Materials and Methods 

Three different thermoplastic polymeric matrices were used, LDPE and PP (both non-polar and semicrystalline) and PS (polar and amorphous). LDPE (PE003 supplied by Repsol Alcudia) has a melt flow index of 2 g/10 min (190 °C and 2.16 kg), 920 kg/m^3^ density, and 110 °C melting temperature. PP (Daploy WB135HMS from Borealis) has a melt flow index of 2.4 g/10min (230 °C, 2.16 kg), 905 kg/m^3^ density, and 165 °C melting temperature. PS (PSC 19060N from TOTAL Refining & Chemicals) has a melt flow index of 30 g/10 min (200 °C, 5.00 kg), and a 1050 kg/m^3^ density. The glass transition temperature of this PS grade is 100 °C. 

Then, LDPE was melt blended with 5 wt.% montmorillonite-type nanoclays: Non-modified sodium montmorillonite clay, Cloisite Na^+^, and two organo-modified clays, Cloisite C20A and Cloisite C30B (Southern Clay Products). These montmorillonites are layered silicates with particle sizes ranging between 2 to 13 microns, according to the technical data sheets. The chemical structures of the organic modifiers (surfactants) used during the organoclay formulation are shown in Table 1 [24]. Nanocomposites of PP and PS were produced using Cloisite Na^+^. To produce the nanocomposites based on LDPE with organo-modified clays, the nanoclays were first mixed with a coupling agent, maleic anhydride grafted polyethylene Fusabond 226 DE from DuPont (melt flow index of 1.5 g/10 min (190 °C, 2.16 kg) and 120 °C melting temperature). The ratio of coupling agent to nanoclays was fixed at 2:1. The rest of the formulations did not include any coupling agent.

LDPE nanocomposites were blended with 7 wt.% ADC (Porofor ADC/M-C1 from Lanxess, Leverkusen, Germany), whereas PP and PS nanocomposites did both with 2.5 wt.% ADC, using a twin screw extruder Collin Teach-Line ZK 25T (Dr. Collin GmbH, Ebersberg, Germany). Blends with ADC, but no clays, were additionally prepared as reference materials. The temperature profiles varied between 105 °C–125 °C (LDPE), 135 °C–155 °C (PP), and 130 °C–150 °C (PS) from the hopper to the die, increasing 5 °C each 10 cm of the extruder. After extrusion, the materials were water cooled, pelletized, then compression-molded into solid sheets of 4 mm in thickness using a two-hot plates press (Talleres Remtex, Barcelona, Spain) at 125 °C, 165 °C, and 150 °C for LDPE, PP, and PS, respectively. From these sheets, foamable precursors of 20 × 10 × 4 mm^3^ were cut. Table 2 summarizes the formulations and nomenclature of all foamable nanocomposites.

PE_0, PP_0, and PS_0 correspond to the reference materials without clays, but maintaining the same percentage of blowing agent as in the materials with clays.

**Figure 1:**
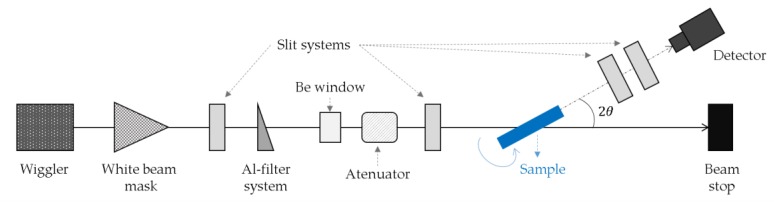
The EDDI beamline at the BESSY II synchrotron light source, adapted from Ref. [25,26,27]. The white beam crosses a system of slits, filters, and attenuators before hitting the sample at the center of the goniometer. The angle 2θ was fixed at 1.7°, all other angles at 0°.

The intercalation of nanoclays during foaming was followed in-situ by ED-XRD at the Energy Dispersive Difraction (EDDI) beamline hosted at the Berlin Electron Storage Ring Society for Synchrotron Radiation (BESSY) II synchrotron light source of the Helmholtz-Zentrum Berlin (Figure 1) [25,26]. Samples were illuminated by a white X-ray beam of a 2 × 1 mm^2^ (height × width) cross-section. Peaks of intensity were detected at particular energies, E_hkl_, in the transmission geometry at an angle of 2θ = 1.7° by a Ge multichannel analyzing detector. The energies of the diffracted photons are related to the interplanar distances, $d_{hkl}$, by the Bragg’s law in its energy-dispersive form (Equation (1)):

(1) $$E_{hkl}=\frac{hc}{2d_{hkl}\sin\theta }$$

where h is Planck’s constant and c the speed of light.

A self-designed X-ray transparent furnace equipped with Si_3_N_4_ heating plates and Kapton windows was mounted on a positioning table attached to a goniometer. The sample size, being 20 × 10 × 4 mm^3^, was placed with the short side (10 mm) parallel to the beam direction and the surface of 20 × 4 mm^2^ perpendicular to it. A thermocouple was placed inside the sample to measure and control the temperature profile using a Novadep temperature controller (Valladolid, Spain) and a self-developed program, which runs under LabView. The temperature was increased from 30 °C to the foaming temperature (200 °C) at a rate of 20 K/min and held there for an isothermal step up to 600 s of the experiment. Then, the heaters were turned off and cooling took place. In situ ED-XRD data acquisition started after 294 s from the beginning of the experiment, when the sample temperature was around 100 °C. The counting time per spectrum was 30 s, after which a lateral sample displacement of 1 mm in the 20 mm sample direction was programed in order to detect each time diffracted photon-energies from a volume, which was previously not irradiated by the X-rays. This displacement step lasted 2 s, so the time interval between spectra acquisition was 32 s. Also, spectra of the samples before heating and after cooling were acquired for 30 s at room temperature. ED-XRD data acquisition and the positioning table were computer controlled by the software package, Spec.

Additional spectra of the three used nanoclays, Na^+^, C20A, and C30B, were also acquired for reference. For each acquired spectrum, the energy corresponding to the maximum of the nanoclays’ peak was fitted using the software, PeakFit (Systat Software, Inc., San José, CA, USA), and then converted into interlamellar spacing using Equation (1). The acquisition of each spectrum lasted 30 s and the midpoint of these 30 s was selected as a representative time for this spectrum. 

The density of the final foams produced in the in-situ experiments was determined by the water-displacement method, based on Archimedes’ principle, using the density determination kit for an AT261 Mettler-Toledo balance (Mettler-Toledo, Columbus, OH, USA). From the density of the foams ($\rho_{f}$) and the density of the solid nanocomposites ($\rho_{s}$), the expansion ratio (ER) was calculated according to Equation (2):

(2) $$ER=\frac{\rho_{s}}{\rho_{f}}$$

Thermogravimetric analysis (TGA) was carried out to evaluate the kinetics of gas release with a TGA equipment, TGA/SDTA 861, from Mettler (Mettler-Toledo, Columbus, OH, USA). 15 mg obtained from the compression molded materials were used for the measurements. The temperature program used was from 50 °C to 1000 °C at a rate of 20 °C/min. From the TGA curve, the onset decomposition temperature of the blowing agent was calculated as the temperature at the intersection of the tangent line to the curve before the decomposition (horizontal line) and the tangent line at the mid-point of the decomposition. At least three TGA experiments per system were performed. The onset temperature was calculated as the average value of these measurements, with the corresponding deviation.

## 3. Results and Discussion

### 3.1. Effects Related to the Type of Clay

The characteristic interlamellar distances of the three types of clays are given in Table 3. The non-modified clay, Na^+^, presents an interlamellar distance of 1.23 nm, whereas the clays, C20A and C30B, have a higher interlamellar distance of around 1.9 nm.

**Figure 2:**
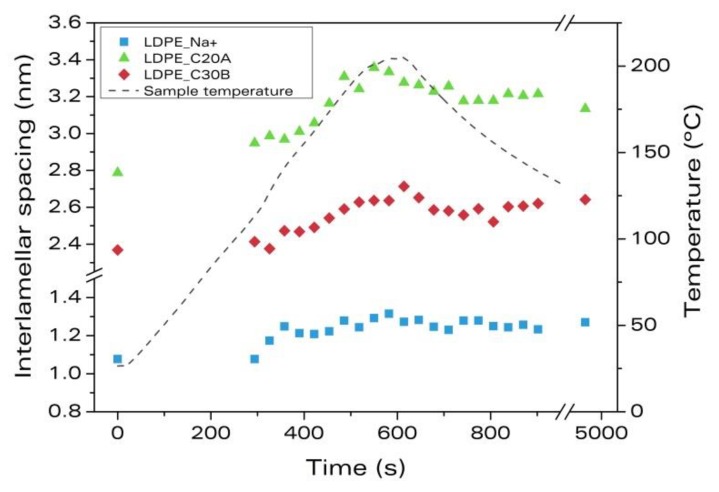
Evolution of the interlamellar spacing of the clay platelets during foaming for the materials based on LDPE.

The evolution of the interlamellar spacing of the LDPE samples as a function of the foaming time is shown in Figure 2 along with the applied temperature profile. The point at time 0 s corresponds to the diffractogram of the solid precursor before foaming, whereas the point at time 4900 s corresponds to the diffractogram of the solidified foam after cooling. 

We associate the modifications of the interlamellar spacing of the platelets with the intercalation of these particles. In order to quantify the change in the interlamellar spacing during the different steps of the materials processing (melt blending and foaming), we define the relative intercalation percentage according to Equation (3):

(3) $$I\left(\%\right)=100\left(\frac{d_{hkl,f}-d_{hkl,0}}{d_{khl,0}}\right)$$

where $d_{hkl,f}$ means the interlamellar spacing at the end of the step considered and $d_{hkl,0}$ is the initial interlamellar spacing, that is, the final spacing of the previous step. On the other hand, the foaming process consists of three stages: Heating and melting of the polymer, expansion of the cells, and cooling and stabilization of the structure.

In the LDPE sample with Na^+^, the interlamellar spacing after melt blending (at time 0 s) is smaller than that of the pure clays (1.076 nm versus 1.23 nm, ca. 12.5% shrinkage (Table 4)). Thus, the melt blending process leads to an aggregation of the Na^+^ nanoclays in this material. Oppositely, the LDPE samples with C20A and C30B nanoclays have a larger interlamellar spacing at the initial time (2.788 nm and 2.367 nm, respectively) compared to the as-received clays (see Table 3 and Table 4). Therefore, for C20A and C30B, the melt blending process helps the intercalation by increasing the interlamellar spacing by 42% and 25%, respectively.

During the first 350 s of the experiment, the temperature increases to above 140 °C. This temperature is smaller than the decomposition temperature of the blowing agent (around 200 °C), so we considered this step as heating and melting of the polymer (Table 4). During this step, interlamellar spacing increases (16%, 7%, and 4% for LDPE_Na^+^, LDPE_C20A, and LDPE_C30B respectively). Thermal expansion of the clays and the polymer matrix might be the reasons for the separation of the nanoclays’ platelets at these temperatures [23].

At around 700 s, the sample temperature reaches the maximum (Figure 2), so we consider the expansion process takes place up to that moment. It is observed that the interlamellar distance further increases with foaming and this increase is a consequence of the foaming process itself, i.e., the volume increase of the sample. The intercalation due to the expansion of the cells process is small for the sample, LDPE_Na^+^ (less than 2%), but significant for the samples, LDPE_C20A and LDPE_C30B (around 10%) (Table 4). 

From this time, the sample temperature starts to decrease (cooling step). The intercalation observed during this process is negative (Table 4), that is, the interlamellar spacing decreases again, probably due to thermal contraction of the foamed sample.

The overall intercalation achieved during foaming (Table 4) shows that intercalation degrees greater than 11% are obtained in the three systems under study. Therefore, the intercalation during foaming occurs for the three systems regardless of the surface modification of the clay.

The foams expansion ratios and densities are also summarized in Table 4. Despite the modified nanoclays showing smaller intercalation in foaming than the non-modified particles, they are intercalated during the melt blending process, so they are more useful to obtain intercalated nanocomposite foams. In addition, LDPE_C20A foam presents the highest expansion ratio.

**Figure 3:**
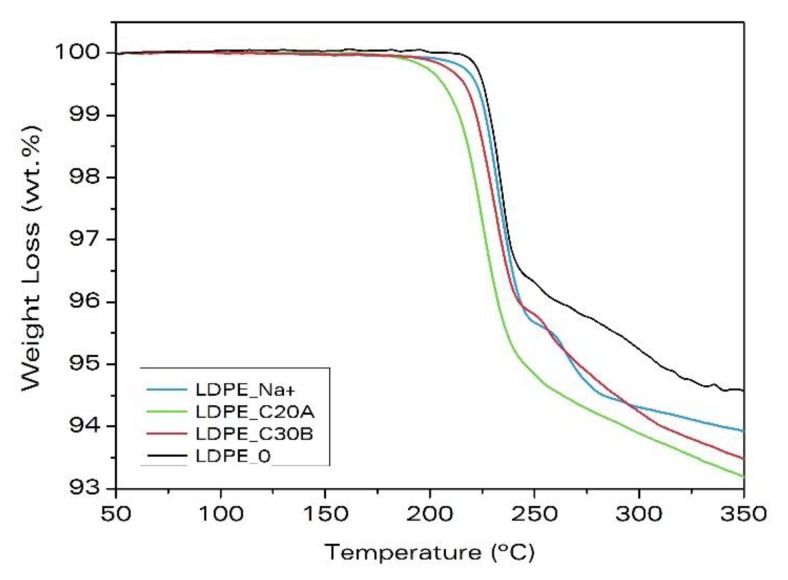
Weight loss measured by thermogravimetry due to the decomposition of the ADC in samples based on LDPE.

The TGA curves for polyethylene samples with and without clays are given in Figure 3. The weight loss is due to the gas released during the decomposition of the ADC. The addition of clays leads to a larger decomposition of the blowing agent. The reference sample, LDPE_0, loses 4.8 wt.% after 750 s, whereas the samples, LDPE_Na^+^, LDPE_C20A, and LDPE_C30B, lose 5.7 wt.%, 6.1 wt.%, and 5.8 wt.%, respectively. Besides, in these samples, the decomposition starts earlier than in the sample without clays, LDPE_0, that is, the onset temperature of the decomposition decreases (Table 5). Therefore, the addition of clays affects the decomposition of the blowing agent, producing a catalytic effect that reduces the onset temperature for decomposition differently depending on the clay-type. It is observed that the addition of clay, C20A, produces the most pronounced effect over the ADC decomposition, by reducing the decomposition temperature by 15 °C and releasing a higher amount of gas than the addition of Na^+^ or C30B. This explains the higher expansion ratio observed in this material.

To further validate that the weight loss measured in Figure 3 is only due to the ADC decomposition, additional TGA analysis of the same formulations, but without the blowing agent (see Table S1), were performed (see Supplementary Information, Figure S1 and Figure S2). Also, the pure clays were tested (Supplementary Information, Figure S3). These experiments support the conclusions already stated.

### 3.2. Effects Related to the Nature of the Polymer

**Figure 4:**
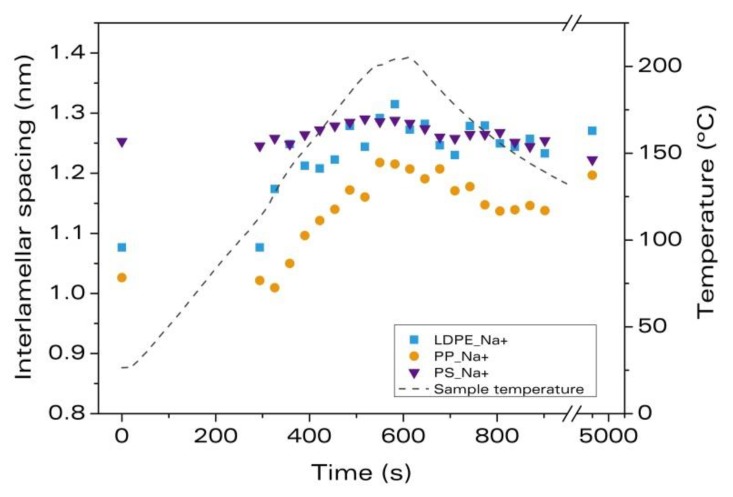
Evolution of the interlamellar spacing of the clay platelets during foaming for the materials with nanoclay, Na^+^.

Figure 4 shows the interlamellar spacing as a function of time and temperature for the polymeric matrices, LDPE, PP, and PS, melt-blended with the non-modified nanoclay, Na^+^.

Regarding the degree of intercalation after melt blending (at t = 0 s), in the material, PP_Na^+^, the separation between platelets at time 0 is smaller than that of the pure clays by almost 17% (see Table 6). This means that the melt-blending process leads to an aggregation of the Na^+^ nanoclays in PP. This result was also observed in Section 3.1 for the sample, LDPE_Na^+^. For the sample, PS_Na^+^, the interlamellar distance at t = 0 s is 1.25 nm, which is 1.9% larger than the spacing of the as-received Na^+^.

Small intercalation (2.3%) is observed for PP_Na^+^ during heating and melting, whereas in PS_Na^+^, the effect is negligible. During the expansion stage, intercalation of the clay platelets takes place for the two systems, but with very different magnitudes: 15% for PP_Na^+^ and 3% for PS_Na^+^. In the cooling step, there is no shrinkage for PP_Na^+^, but in PS_Na^+^, the reduction of the spacing is significant, indicating that the intercalation observed during the foaming process was probably due to the thermal expansion of the polymer and the clays than to the expansion stage itself. Table 6 shows the intercalation percentages reached after each process and the overall intercalation after foaming. It can be concluded that the intercalation during foaming occurs for LDPE and PP, the maximum intercalation being that of LDPE_Na^+^, whereas immeasurable intercalation is found in PS_Na^+^. Therefore, and for this type of clay, there is a clear effect of the type of polymeric matrix on the intercalation during foaming. 

The final densities and expansion ratios reached for each sample are also summarized in Table 6. Similar expansion ratios are obtained regardless of the polymer matrix used. Therefore, the different intercalation degrees are not a consequence of the foam expansion, but are connected with the nature of the interaction between the polymer and the nanoclay.

**Figure 5:**
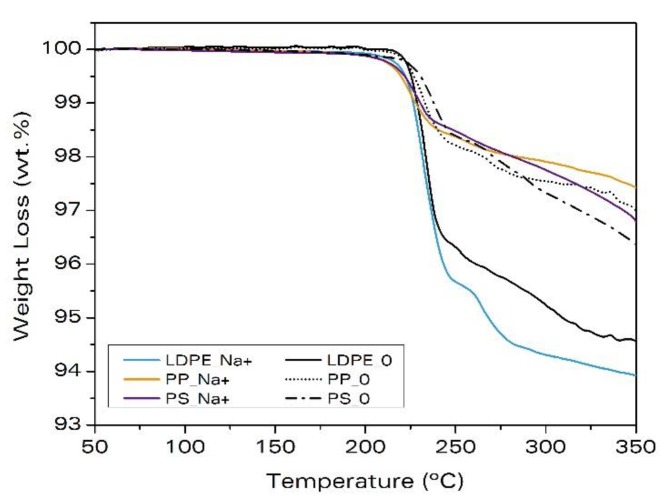
Weight loss measured by thermogravimetry due to the decomposition of the azodicarbonamide in samples with Na^+^.

Figure 5 shows the TGA analysis of these materials. The same as for LDPE_Na^+^, the addition of non-modified clays, Na^+^, to PP and PS causes the decomposition to start earlier than in the samples without clays (Table 7). Therefore, the addition of clays also produces the catalytic effect previously reported by Escudero et al. [23] for LDPE, also in PP and PS.

It should be noticed that the differences in the amount of gas released shown in Figure 5 are due to the different amounts of blowing agent present in the samples.

To further validate that the weight loss measured in Figure 5 is only due to the ADC decomposition, additional TGA analysis of the same formulations, but without the blowing agent, were performed (see Supplementary Information, Figure S1). These experiments support the conclusions already stated.

**Figure 6:**
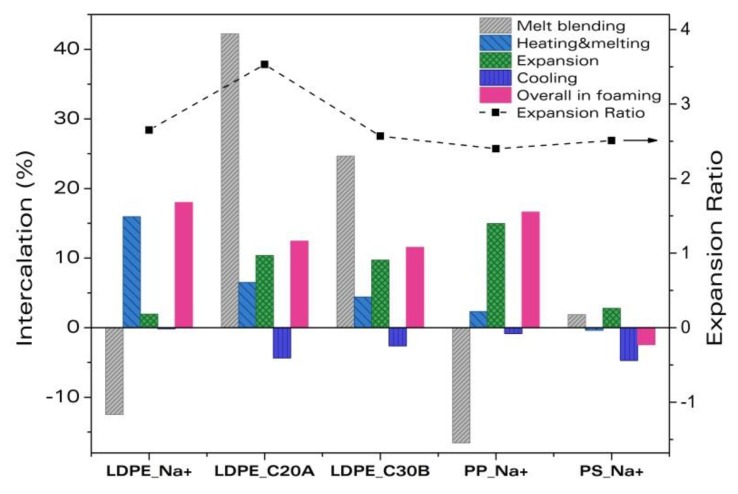
Summary of all the intercalation degrees measured in this work for the different systems and final expansion ratios of the foams.

Figure 6 shows all the intercalation degrees measured in this work along with the final expansion ratios of the foams. It can be observed that the melt blending process makes the largest contribution to the overall intercalation of the clay platelets in those materials with compatibilizer and organo-modified nanoclays. The largest intercalations are observed for LDPE_C20A and LDPE_C30B. This result is widely reported in the literature [22,28,29,30] and is a consequence of the poor chemical interaction between the neat polymer and the non-modified clays, with this interaction being enhanced when the clays are organically modified. The melting of the polymer and the foaming process results in further intercalation of the nanoclays in the PP and LDPE, but not in the PS nanocomposites. Nanocomposites that are best intercalated on foaming do so after undergoing a significant aggregation of clay platelets on melt-blending, these are LDPE_Na^+^ and PP_Na^+^. In PS_Na^+^, the clay platelets aggregate slightly, but consistently, after melt-blending and foaming, resulting in an overall aggregation similar to the one of PP_Na^+^. 

Regarding the expansion of the foams, all the materials present expansion ratios near 2.5 except for the nanocomposite, LDPE_C20A, which shows an expansion ratio larger than 3.5. Thus, LDPE_C20A has both the largest expansion and intercalation degree, making this material the most promising regarding its properties, and is interesting for further studies concerning the aforementioned synergic effect between the ADC and the nanoclays.

The results obtained in this work are useful to clarify the mechanisms of clay intercalation occurring during the foaming process. The intercalation during foaming is similar regardless of the type of clay and the polymer matrix (for LDPE and PP). Also, it depends on the expansion ratio and takes place for different foaming agents (as proved in previous work [23]). All these facts seem to indicate that this intercalation process is a purely physical mechanism related to the separation of the clay platelets due to the extension of the cell walls.

## 4. Conclusions

The effect of the surface modification of the nanoclays in the intercalation of the clay platelets during foaming was investigated using three LDPE-based nanocomposites with different types of clays. Results show that, in these systems, the intercalation during the foaming phenomenon is not associated with the type of clay, but with the foaming process of polyethylene itself. Also, the effect of the polymer matrix was investigated, showing that intercalation during foaming also occurred in PP, but not so much for PS. Therefore, the polymer nature has an important role in this process. Finally, it was proved that the three nanoclays produced a catalytic effect in the decomposition of the blowing agent; that is, in these nanocomposites, the onset of gas release occurs at lower temperatures and the total amount of released gas is larger, with this effect being most pronounced for LDPE_C20A.

From the results of this work, we conclude that the intercalation during foaming taking place in the LDPE and PP systems is a purely physical phenomenon that consists of the separation of the clay platelets due to the extension of the cell walls.

## Figures and Tables

**Table 1:** Surface modifications of the nanoclays used in this work.

Sample Code	Organic Modifier (Surfactant)	Chemical Structure
Na^+^	None	-
C20A	Dimethyl, dihydrogenated tallow, quaternary ammonium	$\left[\begin{array}{ccc} & \begin{array}{c} {\mathrm{CH}}_{3}\\ | \end{array} & \\ H_{3}C- & N^{+} & -\mathrm{HT}\\ & \begin{array}{c} |\\ \mathrm{HT} \end{array} & \end{array}\right]{\mathrm{Cl}}^{-}$
C30B	Methyl, dihydrogenated tallow ammonium	$\left[\begin{array}{ccc} & \begin{array}{c} {\mathrm{CH}}_{2}{\mathrm{CH}}_{2}\mathrm{OH}\\ | \end{array} & \\ H_{3}C- & N^{+} & -T\\ & \begin{array}{c} |\\ {\mathrm{CH}}_{2}{\mathrm{CH}}_{2}\mathrm{OH} \end{array} & \end{array}\right]{\mathrm{Cl}}^{-}$

**Table 2:** Formulations and nomenclature of all foamable nanocomposites used in this work.

Nomenclature	Matrix	Nanoclays	Matrix (Parts)	Coupling Agent (Parts)	Nanoclays (Parts)	Added Blowing Agent (wt.%)
LDPE_Na^+^	LDPE	Na^+^	95	0	5	7
LDPE_C20A	LDPE	C20A	85	10	5	7
LDPE_C30B	LDPE	C30B	85	10	5	7
PP_Na^+^	PP	Na^+^	95	0	5	2.5
PS_Na^+^	PS	Na^+^	95	0	5	2.5
LDPE_0	LDPE	0	100	0	0	7
PP_0	PP	0	100	0	0	2.5
PS_0	PS	0	100	0	0	2.5

**Table 3:** Interlamellar distance of the as-received nanoclays.

Nanoclays	Interlamellar Distance (nm)
Na^+^	1.23
C20A	1.96
C30B	1.90

**Table 4:** Intercalation on melt blending, foaming, and overall intercalation degree, final foam densities, and foam expansion ratios achieved with the different types of nanoclays in the materials based on LDPE.

Sample	At t = 0 s	At t = 358 s	At t = 678 s	At t = 4900 s	Overall Intercalation During Foaming (%)	Foam Density (kg/m^3^)	Foam Expansion Ratio
	Intercalation on Melt Blending (%)	Intercalation During Heating and Melting (%)	Intercalation During Expansion (%)	Intercalation During Cooling (%)			
LDPE_Na^+^	−12.47	15.95	1.93	−0.16	18.01	365	2.7
LDPE_C20A	42.22	6.51	10.37	−4.35	12.44	272	3.5
LDPE_C30B	24.66	4.41	9.71	−2.63	11.54	374	2.6

**Table 5:** Onset temperature of ADC decomposition with the different types of nanoclays in the materials based on LDPE.

Sample	Onset Temperature (°C)
LDPE_C20A	207.4 ± 0.2
LDPE_C30B	216.2 ± 0.4
LDPE_Na^+^	220.3 ± 0.4
LDPE_0	222.1 ± 1.0

**Table 6:** Intercalation on melt blending, foaming, and overall intercalation degree, final foam densities, and foam expansion ratios achieved with the different types of nanoclays in the materials with Na^+^.

Sample	At t = 0 s	At t = 358 s	At t = 678 s	At t = 4900 s	Overall Intercalation During Foaming (%)	Foam Density (kg/m^3^)	Foam Expansion Ratio
	Intercalation on Melt Blending (%)	Intercalation During Heating and Melting (%)	Intercalation During Expansion (%)	Intercalation During Cooling (%)			
LDPE_Na^+^	−12.47	15.95	1.93	−0.16	18.01	365	2.7
PP_Na^+^	−16.56	2.31	14.96	−0.85	16.61	420	2.4
PS_Na^+^	1.87	−0.37	2.78	−4.72	−2.44	416	2.5

**Table 7:** Onset temperature of ADC decomposition with the different types of nanoclays in the materials with Na^+^.

Sample	Onset Temperature (°C)
LDPE_Na^+^	220.3 ± 0.4
LDPE_0	222.1 ± 1.0
PP_Na^+^	213.3 ± 0.3
PP_0	221.0 ± 0.6
PS_Na^+^	215.2 ± 0.2
PS_0	226.6 ± 0.1

## Supplementary Materials

The following are available online at http://www.mdpi.com/1996-1944/11/12/2459/s1, Figure S1: Weight loss measured by thermogravimetry in the samples based on LDPE, with blowing agent and without it (w/oADC). a) LDPE_0, b) LDPE_Na^+^, c) LDPE_C20A and d) LDPE_30B, Figure S2: Weight loss measured by thermogravimetry in the samples with Na^+^, with blowing agent and without it (w/oADC). a) PP_0, b) PP_Na^+^, c) PS_0 and d) PS_Na^+^, Figure S3: Weight loss measured by thermogravimetry in the as-received nanoclays, Table S1: Formulations and nomenclature of the nanocomposites without blowing agent.
