# Supplementary material for: Nanoclay Intercalation During Foaming of Polymeric Nanocomposites Studied in-Situ by Synchrotron X-Ray Diffraction
Source: Materials (Basel). 2018 Dec 4;11(12):2459. doi: 10.3390/ma11122459 (PMC6317266; doi:10.3390/ma11122459)
Supplement: Supplementary file 1 [file materials-11-02459-s001.pdf]

materials

MDPI

*Supplementary Material*

# Nanoclay Intercalation During Foaming of Polymeric Nanocomposites Studied in-Situ by Synchrotron X-Ray Diffraction

**Victoria Bernardo [1,*], Mikel Mugica [1], Saul Perez-Tamarit [1], Belen Notario [1], Catalina Jimenez [2] and Miguel Angel Rodriguez-Perez [1]**

[1] Cellular Materials Laboratory (CellMat), Condensed Matter Physics Department, University of Valladolid, Paseo de Belen 7, 47011 Valladolid, Spain; mikel.mugica@fmc.uva.es (M.M.); saul.perez@fmc.uva.es (S.P.-T.); belen.notario@fmc.uva.es (B.N.); marrod@fmc.uva.es (M.A.R.-P.)

[2] Helmholtz-Zentrum Berlin, Hahn Meitner Platz 1, 14109 Berlin, Germany. catalina.jimenez@helmholtz-berlin.de

* Correspondence: vbernardo@fmc.uva.es; Tel: +34 983423194

## S1. TGA analysis of the materials without blowing agent

Thermogravimetric analysis (TGA) was carried out in the formulations without blowing agent. This analysis was done with the aim of assuring that the weight loss associated with the decomposition of the blowing agent in Figure 3 and 5 was mainly due to the decomposition of the ADC. Table S1 summarizes the nomenclature of the nanocomposites produced for this study. Note that the proportions are the same as those in Table 1, but without adding the chemical blowing agent.

**Table S1.** Formulations and nomenclature of the nanocomposites without blowing agent.

| Nomenclature | Matrix | Nanoclays | Matrix (Parts) | Coupling Agent (Parts) | Nanoclays (Parts) | Blowing Agent (wt.%) |
|---|---|---|---|---|---|---|
| LDPE_Na+_w/oADC | LDPE | Na+ | 95 | 0 | 5 | 0 |
| LDPE_C20A+_w/oADC | LDPE | C20A | 85 | 10 | 5 | 0 |
| LDPE_C30B+_w/oADC | LDPE | C30B | 85 | 10 | 5 | 0 |
| PP_Na++_w/oADC | PP | Na+ | 95 | 0 | 5 | 0 |
| PS_Na++_w/oADC | PS | Na+ | 95 | 0 | 5 | 0 |
| LDPE_0+_w/oADC | LDPE | 0 | 100 | 0 | 0 | 0 |
| PP_0+_w/oADC | PP | 0 | 100 | 0 | 0 | 0 |
| PS_0+_w/oADC | PS | 0 | 100 | 0 | 0 | 0 |

Figure S1 shows the TGA analysis of the formulations without blowing agent based on LDPE. For the sake of comparison, the TGA of the same material with the blowing agent has been included. In Figure S1.a the decomposition of a LDPE sample without ADC and without clays is presented (LDPE_0_w/oADC). It is observed that LDPE does not suffer any decomposition in the range of temperatures at which the decomposition of the ADC starts. In the sample with Na+ but without ADC (Figure S1.b) there is a small decomposition of less than a 0.5 wt.%, probably due to the decomposition of organic compounds in the surface of the clays. However, this drop in the TGA curve starts at a much lower temperature than the ADC decomposition (100 °C versus 220 °C), so it cannot affect our determination of the onset temperature. On the other hand, in the samples with C20A and C30B without ADC no decomposition is observed in the range of onset temperatures, so we conclude that the coupling agent and the clays are not influencing our determination of the ADC onset decomposition temperature. Thus, the onset temperatures calculated in Table 5 are ascribed only to thermal decomposition of ADC.

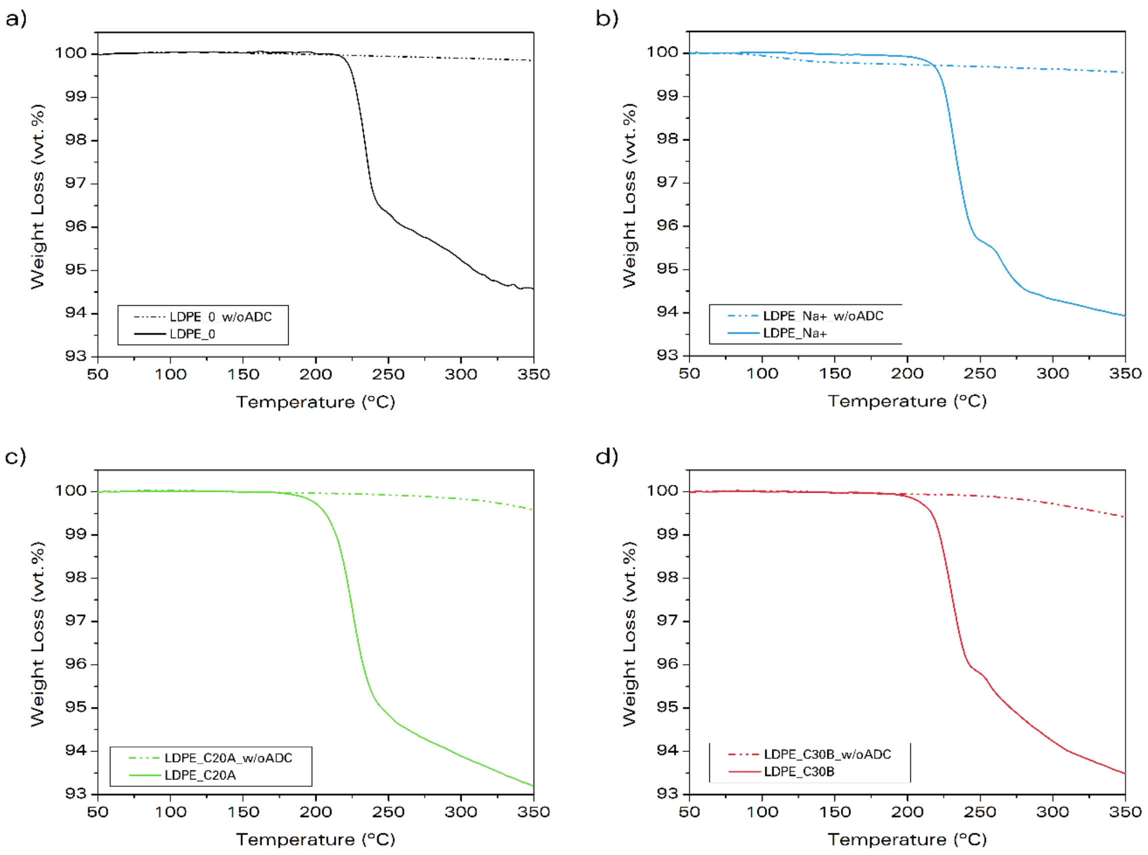


**Figure S1.** Weight loss measured by thermogravimetry in the samples based on LDPE, with blowing agent and without it (w/oADC). a) LDPE_0, b) LDPE_Na+, c) LDPE_C20A and d) LDPE_30B.

Similar trends are observed for the materials with Na+ based on PP and PS. Figure S2 shows the TGA analysis of the samples based on PP and PS, both with clays and without clays, and with and without blowing agent. We observe that none of the samples without blowing agent shows a decomposition around the ADC decomposition temperature. Therefore, we claim that the onset temperatures calculated in Table 7 are ascribed only to thermal decomposition of ADC.

## S2. TGA analysis of the raw nanoclays

Finally, thermogravimetric analysis (TGA) of the as-received nanoclays were performed. Before the analysis, the clays were dried to remove the moisture. Figure S3 shows the result of this analysis for the three nanoclays. Note that even though the TGA was carried out until 1000 °C, only the region of interest for comparison with the nanocomposites (until 350 °C) is plotted. For the Na+, we see that there is a small decomposition at the beginning of the curve of around 1.5 wt.% (1.41 wt.% at 200 °C, that is, the foaming temperature). Given the fact that the formulations of this work have 5 parts of clays, the decomposition of the pure clay would represent 0.07 wt.% in the nanocomposites without ADC. In the case of the clays with surface modification, the weight loss is significantly higher over the temperature range due to the surface compounds, but in any case, it still represents a small fraction in the nanocomposites. In fact, at the foaming temperature (200 °C) in the clay C20A, the weight lost is 0.45 wt.%, that is, 0.02 wt.% in a nanocomposite with 5 parts of clays and without ADC. In the clay C30B, this loss is 0.85 wt.%, which represents 0.04 wt.% in the nanocomposites (the fractions calculated would be slightly smaller in the samples with ADC). In any case, the mass lost in the pure clays is small once we take into account the fraction of particles in the solid nanocomposites. In fact, in Figure S1 and Figure S2 this weight loss was not appreciated at all, but slightly in Figure S1.b. Therefore, we conclude that the mass lost in the clays is not affecting our calculation of the onset temperature and the conclusions of this work.

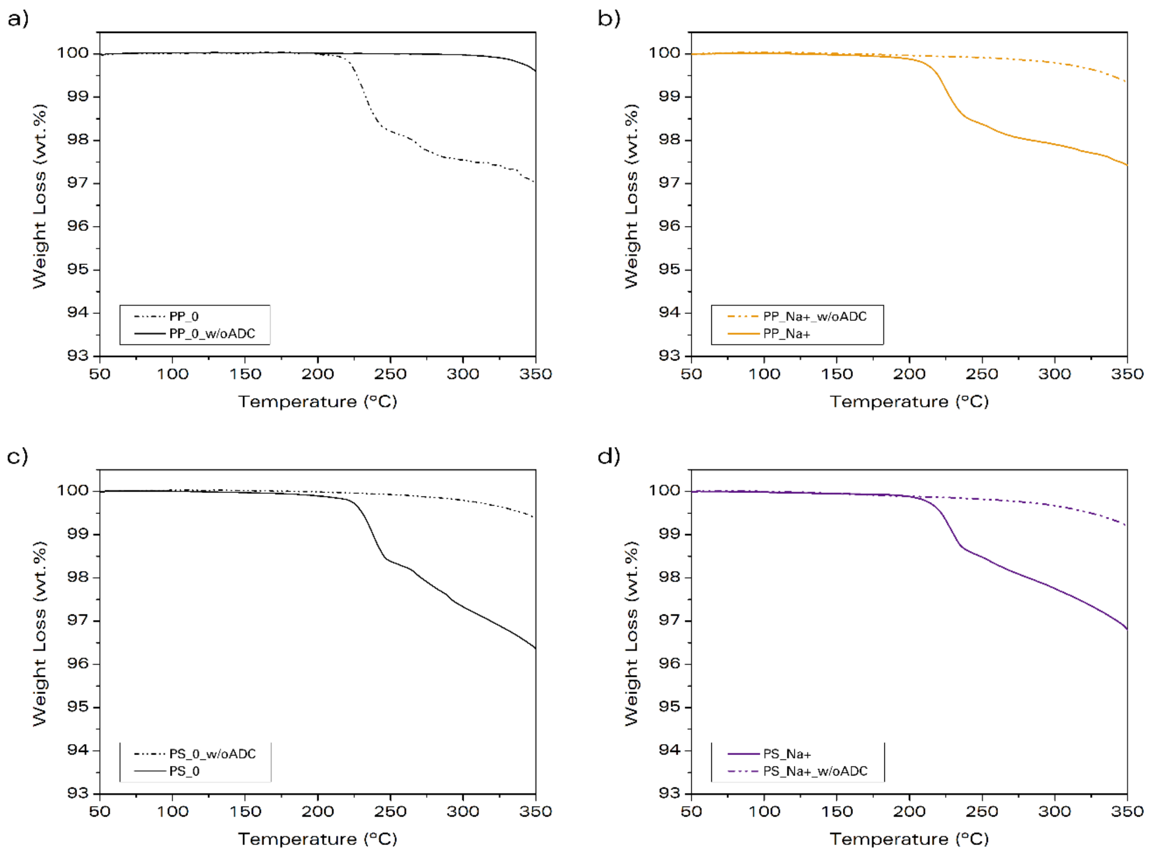


**Figure S2.** Weight loss measured by thermogravimetry in the samples with Na+, with blowing agent and without it (w/oADC). a) PP_0, b) PP_Na+, c) PS_0 and d) PS_Na+.

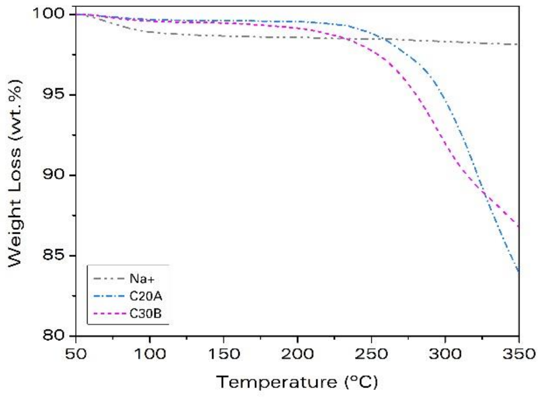


**Figure S3.** Weight loss measured by thermogravimetry in the as-received nanoclays.
